# Nerve Fibres in Psoriatic Skin and Their Relation to Vasculature and Clinical Parameters

**DOI:** 10.1111/exd.70166

**Published:** 2025-09-24

**Authors:** Meagan Doppegieter, Nick van der Beek, Maurice C. G. Aalders, Erik N. T. P. Bakker, Martino Neumann, Ton G. van Leeuwen

**Affiliations:** ^1^ Biomedical Engineering and Physics Amsterdam UMC, University of Amsterdam Amsterdam the Netherlands; ^2^ Amsterdam Cardiovascular Sciences Amsterdam the Netherlands; ^3^ ZBC Multicare Hilversum the Netherlands; ^4^ Personalized Medicine Amsterdam Public Health Amsterdam the Netherlands; ^5^ Neurovascular Disorders Amsterdam Neuroscience Amsterdam the Netherlands; ^6^ Heart Failure and Arrhythmias Amsterdam Cardiovascular Sciences Amsterdam the Netherlands; ^7^ Atherosclerosis & Ischemic Syndromes Amsterdam Cardiovascular Sciences Amsterdam the Netherlands; ^8^ Imaging and Biomarkers Cancer Center Amsterdam Amsterdam the Netherlands

## Abstract

Emerging evidence supports the neurogenic origin of psoriasis, yet the morphology and distribution of nerve fibres in psoriatic skin remain poorly characterised due to methodological inconsistencies and limited 3D data. The aim of this study was to provide a comprehensive 3D quantification of nerve fibre morphology in psoriatic skin and assess its spatial relation to vasculature and clinical parameters. High‐resolution confocal microscopy was used to analyse 69 (70 μm thick) skin sections from 23 psoriasis patients, capturing full‐thickness epidermis and dermis. Nerve fibres were segmented by location (epidermal, papillary and reticular) and quantified volumetrically alongside vascular networks. The results show that nerve fibres occupied ~0.1% of total skin volume and predominantly localised near vasculature in the dermis, with epidermal nerves branching from perivascular plexuses. Epidermal nerve fibre volume negatively correlated with erythema, age and epidermal thickness (*p* < 0.05). No significant correlation was observed between dermal nerve fibre volumes and vascular density or clinical severity scores. This study presents a detailed 3D neurovascular map of psoriatic skin, revealing a distinct topography of nerve‐vessel relationships. The findings highlight that epidermal nerve fibres (not total nerve density) show the strongest association with clinical markers. These results provide a critical baseline for evaluating nerve‐targeted therapies and modelling neurovascular responses in laser‐based psoriasis treatments.

## 1 Introduction

Psoriasis is a chronic inflammatory skin disorder characterised by aberrant keratinocyte proliferation, immune dysregulation and prominent microvascular remodelling. While most research has focused on immunological pathways, there is increasing recognition of the nervous system as a critical, yet understudied, contributor to psoriatic pathogenesis. Clinical observations, such as lesion exacerbation during stress and experimental findings implicating neuropeptides in vasodilation, mast cell degranulation and keratinocyte hyperproliferation, point toward a neurogenic component of inflammation [1, 2, 3, 4, 5, 6, 7, 8].

The concept of neurogenic inflammation in psoriasis is not new. As early as 1955, Szodoray hypothesised a neural influence [9], later substantiated by Farber and others who demonstrated stress‐mediated exacerbation and neuropeptide involvement [1, 2]. Sensory nerve endings in the skin release mediators such as CGRP, substance P and VIP, which modulate vascular tone, leukocyte recruitment and keratinocyte activity; core features of psoriatic lesions [7, 10, 11, 12].

Despite this, the morphological characterisation of nerves in psoriatic skin remains inconclusive. Previous studies have reported conflicting findings, with some noting increased innervation [13, 14] and others a decreased [15, 16] innervation, probably largely due to methodological inconsistencies such as variable section thicknesses, imprecise depth stratification and 2D analysis.

Most importantly, earlier work often ignored the spatial relationship between nerves and other key components of psoriatic pathology, including vasculature and immune infiltrates. Without this spatial context, the functional relevance of nerve morphology remains speculative.

A 3D morphological analysis across all skin layers is lacking, yet essential. Understanding how nerve fibres are distributed relative to vessels and how their density correlates with clinical presentation could provide mechanistic insights and improve biomarker identification or therapeutic targeting. Additionally, given that certain laser and light‐based therapies may act via neuromodulation, baseline nerve architecture in psoriasis must be established [17, 18].

In this study, we performed high‐resolution 3D imaging of full‐thickness psoriatic skin biopsies from 23 patients to map nerve fibre distribution in the epidermis, papillary dermis and reticular dermis. We quantitatively assessed the spatial relationship of nerves to vasculature and evaluated how nerve densities relate to clinical parameters, including erythema, plaque thickness, itch and overall disease severity. This work aims to clarify the structural neurovascular landscape of psoriatic skin and assess whether morphological nerve patterns mirror clinical markers such as erythema, scaling, induration and PGA.

## 2 Materials and Methods

### 2.1 Study Design and Participants

We performed a biopsy study to evaluate the correlation between clinical assessment and histopathology of skin tissue, with a special focus on the neurovascular anatomy of the skin. The study was conducted between December 2022 and April 2024. Eligible patients were adults (18–69 y/o) with psoriasis and Fitzpatrick skin type I–III. Exclusion criteria were pregnancy and known neurological conditions. In total, 23 patients were included. This study was conducted in accordance with the protocol and consensus ethical principles derived from international guidelines including the declaration of Helsinki and the ICH guidelines for Good Clinical Practice (GCP), approved by the Ethics committee of the Medical Ethical Committee of MEC‐U, Nieuwegein, The Netherlands. All study participants signed the informed consent form prior to participation in the study.

### 2.2 Clinical Assessment and Sampling

Patients were asked for weight in kg, length in cm, smoking status (smoker, stopped smoking, non‐smoker) and itch. The clinicians scored the study plaques for erythema (0–4), scaling (0–4) and induration (0–4). An external expert scored the photographs for the Physician Global Assessment (PGA), which is a commonly accepted score for the severity of psoriasis. Scores were classified with 1 being non‐existent and 7 being a severe psoriasis lesion. Three‐millimetre punch biopsy specimens were obtained from psoriatic skin lesions under local anaesthesia of 1% Xylocaine.

### 2.3 Immunohistochemistry

Skin sections were labelled for a junctional protein of endothelial cells (CD31), nerve fibres (PGP9.5) and cell nuclei (Dapi, Hoechst). To do so, biopsies were fixed upon arrival for 24 h in Zamboni Fixative at 4°C, washed twice in Sorrenson's buffer and stored at 4°C in a 20% glycerol buffer for 24 to 72 h. Then, tissues were embedded in OCT TissueTek (Sakura Finetek Europe, Alphen aan den Rijn, The Netherlands) and sectioned at 80 μm with a Cryostat Cryostar NX70 (Thermo Scientific). Free‐floating 80 μm thick skin sections were submerged in antifreeze buffer and stored at −20°C in round bottom 96‐well plates, until used. The staining protocol consisted of a 10 min wash in dPBS, followed by a 2 h blocking step in 4% normal goat serum (Agilent Technologies Singapore, X0907) and 0.1% triton X‐100 in dPBS to permeabilise the tissue. Sections were then incubated with 1:500 mouse anti‐human CD31 conjugated to AF647 (BD pharmingen, 561 654) and 1:300 rabbit anti‐human PGP9.5 (Abcam, ab108986) in a 1:1 ratio of dPBS and blocking solution at 4°C overnight. Sections were rinsed twice in dPBS for 5 min and then incubated with 1:500 goat anti‐rabbit Cy3 (Jackson Lab, 115–165‐044) and 1:1000 Hoechst (0.1 mg/mL, 33 342) diluted in dPBS for 2 h on a mild shaker at RT. To prevent photobleaching, the container lid was covered with aluminium foil. Then, slides were rinsed in MilliQ to remove salt crystal formation and mounted in Dapi‐containing Vectashield (Vector Laboratories, H‐1200‐10).

### 2.4 Imaging and Histology

Three non‐consecutive sections from each patient were imaged using an SP8‐X DLS Lightsheet confocal microscope (Leica) with an HC PL APO 40X/1.30 OIL CS2 objective. For each section, *Z*‐stacks were captured with dimensions of 1.1 mm × 1.6 mm × 70 μm (xyz), using a 0.5 μm *z*‐step size. Laser power was optimised for each patient and maintained consistently across sections for that individual. Nerve fibre volume was calculated using IMARIS software (Oxford Instruments, version 10.1) as the relative volume (%) of fluorescently stained nerve fibres (green) compared to the total skin volume. Nerves were classified based on their location in the epidermis, papillary dermis or reticular dermis. Similarly, blood vessel volume (red) was measured in the same manner. Blood vessels were classified as either papillary vasculature or reticular vasculature. Total skin volume was determined by overexposing the blue channel.

To evaluate scaling, we measured the thickness (μm) of the hyperparakeratotic layer, and epidermal thickness was determined from the skin surface to the base of the rete ridges (epidermal extensions reaching from the surface to the dermo‐epidermal junction). Five measurements were taken per section, across three sections (yielding 15 measurements per biopsy) and averages with standard deviations were calculated.

### 2.5 Statistics

The data are presented as mean ± standard deviation (SD). To compare two groups, we used an unpaired t‐test. For comparison among three or more groups, One‐Way ANOVA testing was used with a Tukey test for multiple comparisons. For correlation calculations, we used Pearson *r* statistics. Correlation graphs show the Pearson *r* value unless otherwise indicated. *p*‐values were considered significant at a value of 0.05. All statistical analyses were performed with GraphPad Prism software (Graphpad, Boston, USA, version 9.5.1).

## 3 Results

### 3.1 General Study Population

**FIGURE 1:**
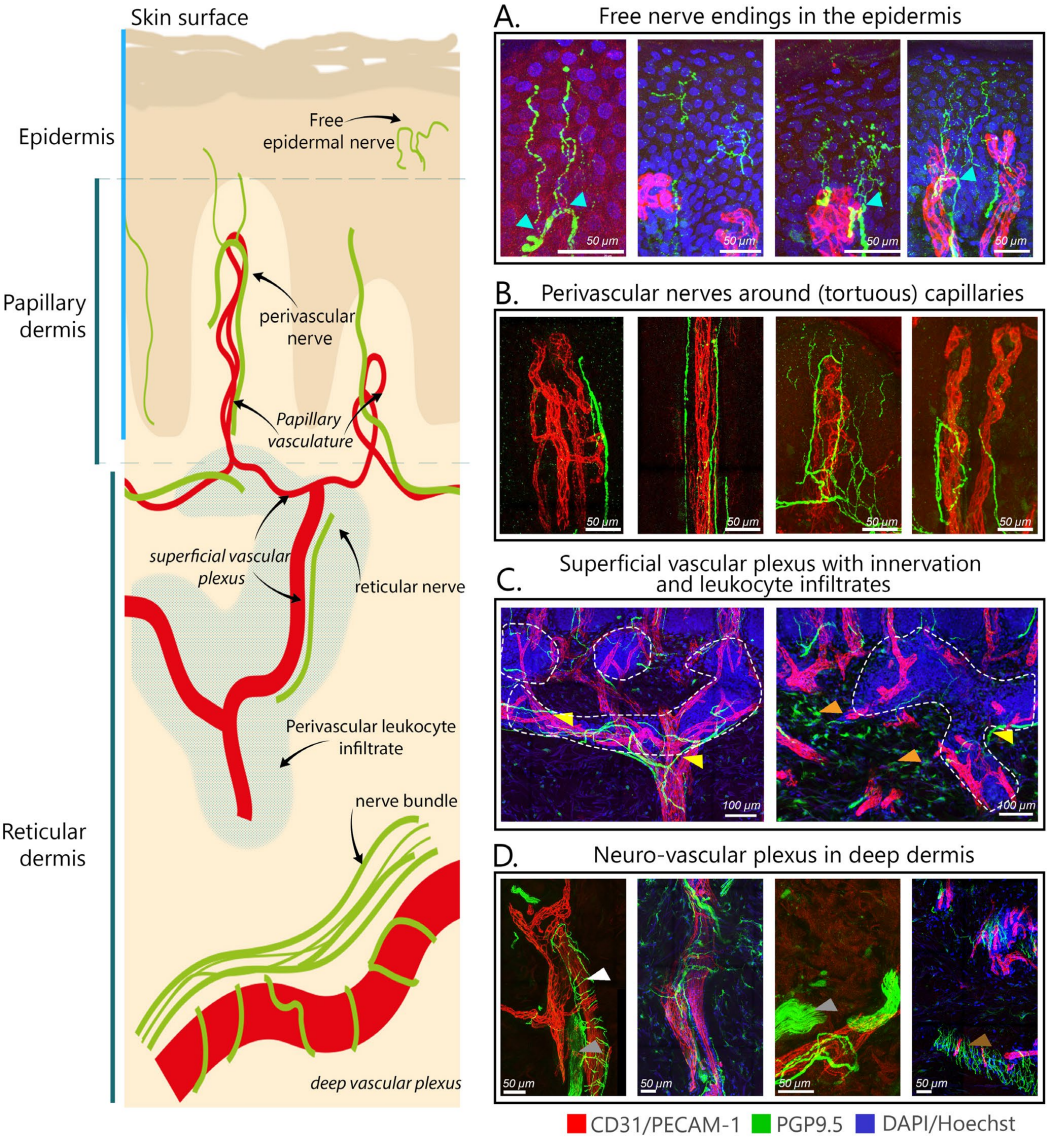
Neurovascular architecture in psoriatic skin, highlighting nerve and vascular interactions across epidermal, papillary and reticular dermal layers. Left panel: Schematic representation of psoriatic skin layers, showing the distribution of free nerve endings (green) and vascular structures (red) in the epidermis, papillary dermis and reticular dermis. (A) Free nerve endings (green) in the epidermis that split off from perivascular nerves in the papillae. (B) Papillary perivascular nerves surrounding capillaries in the papillary dermis, (C) Superficial reticular vascular plexus with accompanying nerve fibres (yellow arrows) and leukocyte infiltrates (line drawing), and pgp9.5+ fibroblasts (orange arrows). (D) Neurovascular plexus in the deep reticular dermis, showing thick nerve bundles (grey arrow) running parallel to deep vascular plexus and fine nerve fibres surrounding the vasculature (arteries) (white arrows). Orange arrows show pgp9.5+ fibroblasts in some patients but not in all. Brown arrow shows innervation typically seen at the erector pili near hair follicles.

A total of 23 participants were included in this study. Biopsies were taken from the elbows (*n* = 10), trunk (*n* = 8) and the extremities (*n* = 5) (Table 1). Images of the corresponding plaques are shown in Figure 1; sometimes the location of the biopsy is also demarcated. The average age was 44 ± 13 years (Table 1). The mean BMI of the participants was 25.9 ± 4.0, of which 43% (10/23) had a normal BMI equal to or less than 24.9, 39% (9/23) were overweight (BMI between 25.0 and 29.9), and 17% were obese (BMI > 30). The Physician Global Assessment (PGA) score indicated that the mean severity of psoriasis was 3.7 ± 1.38. PGA did not correlate with BMI or age, but age correlated significantly with BMI. The PGA score was significantly higher in smokers than in people who did not smoke at the moment of inclusion. Epidermal thickness was significantly lower in patients that had family members with psoriasis (*p* > 0.05). All plaques are shown in Figure S1. Correlation plots of the parameters are shown in Figure S2.

**TABLE 1:** Patient descriptives.

Patient	Skin type	Location	Genetic	Gender	Age	Smoking	BMI	Itch (0–4)	PGA (1–7)
**1**	3	Elbow	Yes	Male	43	Yes	22.6	1	6
**2**	2	Upper Leg	Yes	Female	60	Stopped	25.5	2	3
**3**	3	Elbow	No	Male	35	No	26.6	4	4
**4**	2	Lower Arm	Yes	Male	29	Stopped	23.7	3	3
**5**	2	Hip	No	Male	53	Yes	32.6	1	3
**6**	2	Elbow	No	Male	38	Yes	21.2	2	4
**7**	2	Elbow	Yes	Male	63	No	34.1	3	2
**8**	2	Hip	No	Male	39	Stopped	29.3	3	4
**9**	2	Trunk	Yes	Male	41	Yes	25.0	1	4
**10**	2	Hip	Yes	Male	60	Stopped	28.8	2	2
**11**	2	Hip	Yes	Male	45	Stopped	30.9	2	3
**12**	2	Hip	No	Male	68	Stopped	26.2	1	3
**13**	2	Back	No	Male	31	Yes	21.0	2	3
**14**	2	Upper Legs	No	Male	54	N.A.	28.6	4	5
**15**	2	Elbow	No	Male	35	Stopped	23.5	0	4
**16**	2	Upper Legs	Yes	Male	40	No	23.8	2	2
**17**	3	Elbow	No	Female	19	Yes	19.8	4	6
**18**	3	Elbow	No	Female	40	Stopped	22.1	0	5
**19**	2	Elbow	Yes	Male	46	Stopped	23.9	2	4
**20**	1	Elbow	Yes	Male	54	N.A.	30.4	1	5
**21**	2	Elbow	No	Male	61	Yes	29.3	2	7
**22**	2	Back	Yes	Female	25	Stopped	21.2	2	2
**23**	2	Upper Arm	No	Male	33	Stopped	26.2	0	3
Mean ± SD					44 ± 13		26 ± 4	1.9 ± 1.2	3.8 ± 1.4

### 3.2 Histological Evaluation of the Neurovascular Anatomy in Psoriatic Skin

**FIGURE 2:**
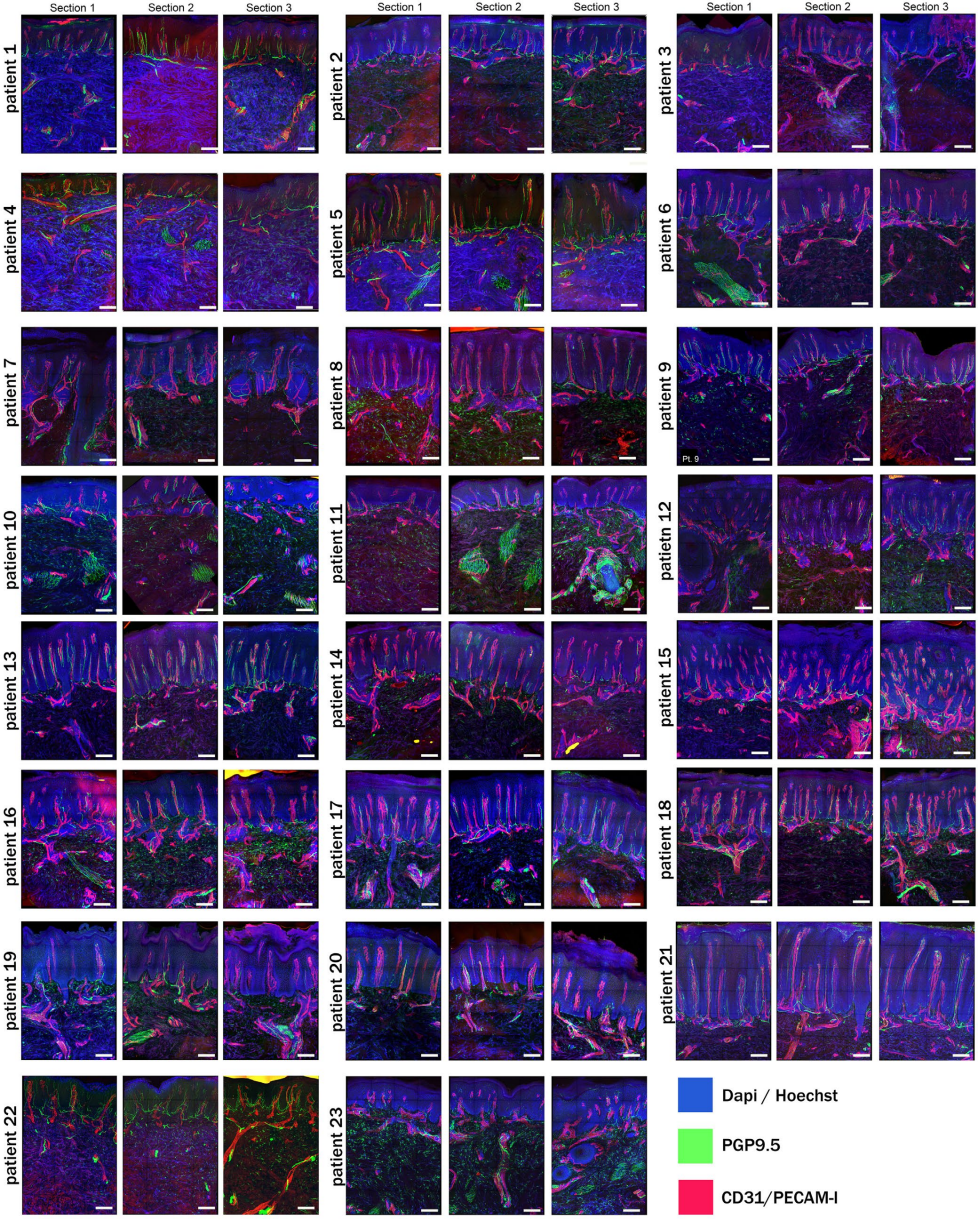
Immunofluorescent histological images of skin sections, oriented with the epidermis at the top and the reticular dermis at the bottom. The psoriatic vasculature, characterised by tortuous and elongated vessels, is visible in red. Thin green fibres indicate (peri)vascular nerves, including those innervating the erector pili muscles (patient 6 S1, patient 11 S2) and the hair follicle (patient 11 S3). Variations in epidermal thickness are also evident, with vertical vasculature marking the transition between the epidermis and the horizontal vasculature of the reticular dermis.

Nerve fibres were present in the epidermis, papillary dermis and reticular dermis where they tended to have distinct morphologies. Figure 1 illustrates an overview in which the most common characteristics of the innervation and its relation to the vasculature are shown, whereas Figure 2 shows all individual skin sections stained for nerve fibres (PGP9.5) and blood vessels (CD31) per patient. The mean percentage over the entire study cohort of epidermal nerves was found to be 0.003% ± 0.008%, while the mean percentage of papillary nerves was 0.09% ± 0.05%, and the mean percentage of reticular nerves was 0.1% ± 0.06%. In addition, the collective total percentage of nerves within the psoriatic skin section was 0.09% ± 0.05%.

**FIGURE 3:**
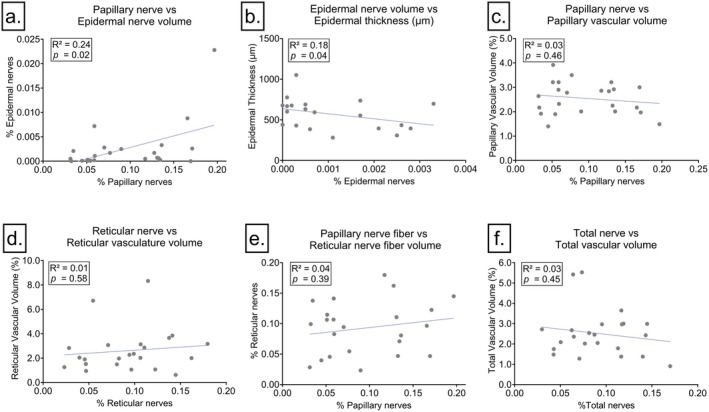
Correlation analyses of nerve fibre and vascular volume percentages across different skin layers in psoriatic tissue sections*.* (a) Papillary nerve fibre volume positively correlates with epidermal nerve fibre volume (*R* ^2^ = 0.24, *p* = 0.02). (b) A significant *inverse relationship is observed between epidermal* nerve fibre volume and epidermal thickness (*R* ^2^ = 0.18, *p* = 0.04). (c) No significant correlation is found between papillary nerve fibre volume and papillary vascular volume (*R* ^2^ = 0.03, *p* = 0.46). (d) Reticular nerve fibre volume shows *no significant correlation* with reticular vascular volume (*R* ^2^ = 0.01, *p* = 0.58). (e) *Comparison of papillary nerve fiber volume with reticular* nerve fiber volume shows no significant correlation (*R* ^2^ = 0.04, *p* = 0.39. (f) The total nerve volume does not correlate significantly with *total vascular volume (R* ^2^ *= 0.03, p =* 0.45).

Morphologically, epidermal nerve fibres varied in branching complexity and tortuous course. We observed in some sections that epidermal nerve fibres branched from the perivascular nerves surrounding the capillaries (Figure 1a). Statistical testing confirmed the observation that epidermal nerves branched from papillary nerves by showing a significant relationship between papillary nerve fibres and epidermal nerve fibres (*R* ^*2*^ *= 0.24. p = 0.02*, Figure 3a *)*. Epidermal nerve fibre volume also correlated significantly with epidermal thickness (*R* ^*2*^ *= 0.18, p = 0.04*, Figure 3b *)*. Perivascular nerves were found abundantly surrounding the capillaries in the papillary epidermis (Figure 1b). The majority of perivascular nerves were observed to run parallel with the capillaries. However, there was a notable variation in the network density of the fibres among patients, with some exhibiting a single nerve fibre running through the capillaries and others displaying a full mesh network of fibres. The capillaries observed in the papillary dermis exhibited the typical tortuosity and elongation extending to a considerable depth within the epidermis. However, it should be noted that the degree of tortuosity observed among these capillaries varied between patients. There was no relationship between the papillary blood vessel volume (%) and the perivascular nerve fibres in the papillae (%) (*R* ^*2*^ *= 0.03*, *p* = 0.46, Figure 3c), indicating that more perivascular nerves do not infer more blood vessels, or *vice versa*. Directly beneath the papillary dermis, we frequently observed perivascular leukocyte infiltrates surrounding the supplying blood vessels (Figure 1c, Figure S3) which were often accompanied by (perivascular) nerve fibres. We also observed fibroblast‐shaped cells positive for nerve staining in the upper and lower reticular layers, although not in all patients (Figure 1c). Toward the lower reticular dermis, we observed larger supplying blood vessels that were sometimes wrapped in unmyelinated nerve fibres, crossing thicker nerve bundles or wavy patterns of nerve fibres that are characteristic of the innervation of erector pili muscles (Figure 1d). There was no significant correlation between nerve density in the reticular dermis and blood vessel volume in the reticular dermis (*R* ^*2*^ *=* 0.01, *p* = 0.58). The papillary nerve volume did not correlate to the reticular nerve fibre volume (*R* ^*2*^ = 0.04, *p* = 0.39), suggesting that the density of nerve fibres in the papillary dermis is not because of a high density of reticular ‘supplying’ nerve fibres. In blood vessels, we did see a correlation between papillary blood vessel volume and the ‘supplying’ reticular blood vessel volume of *r* = 0.5 (*p* = 0.017). Total nerve fibre volume did also not correlate with total vascular volume (Figure 3f).

### 3.3 Patient‐Specific Variation in Nerve Fibre Density

To assess the reliability and biological relevance of nerve fibre quantification, we analysed both within‐biopsy (intra‐sample) and between‐patient (inter‐sample) variability. Our findings demonstrate that the vast majority of variation in nerve fibre density arises at the inter‐patient level, while differences between sections within the same biopsy were minimal and statistically non‐significant (Table S1). This suggests that our sampling and imaging approach yields reproducible data and that observed variability is reflective of true biological heterogeneity rather than technical noise.

### 3.4 Nerve Density and Clinical Parameters

Given that we established in the previous paragraph that there is a significant discrepancy in nerve fibre density between patients, we proceeded to investigate whether variation in nerve fibre density could be explained by a correlation with clinical parameters. These parameters included itch, erythema, scaling, thickness, Physician Global Assessment, BMI, age, smoking status and location of the biopsy.

**FIGURE 4:**
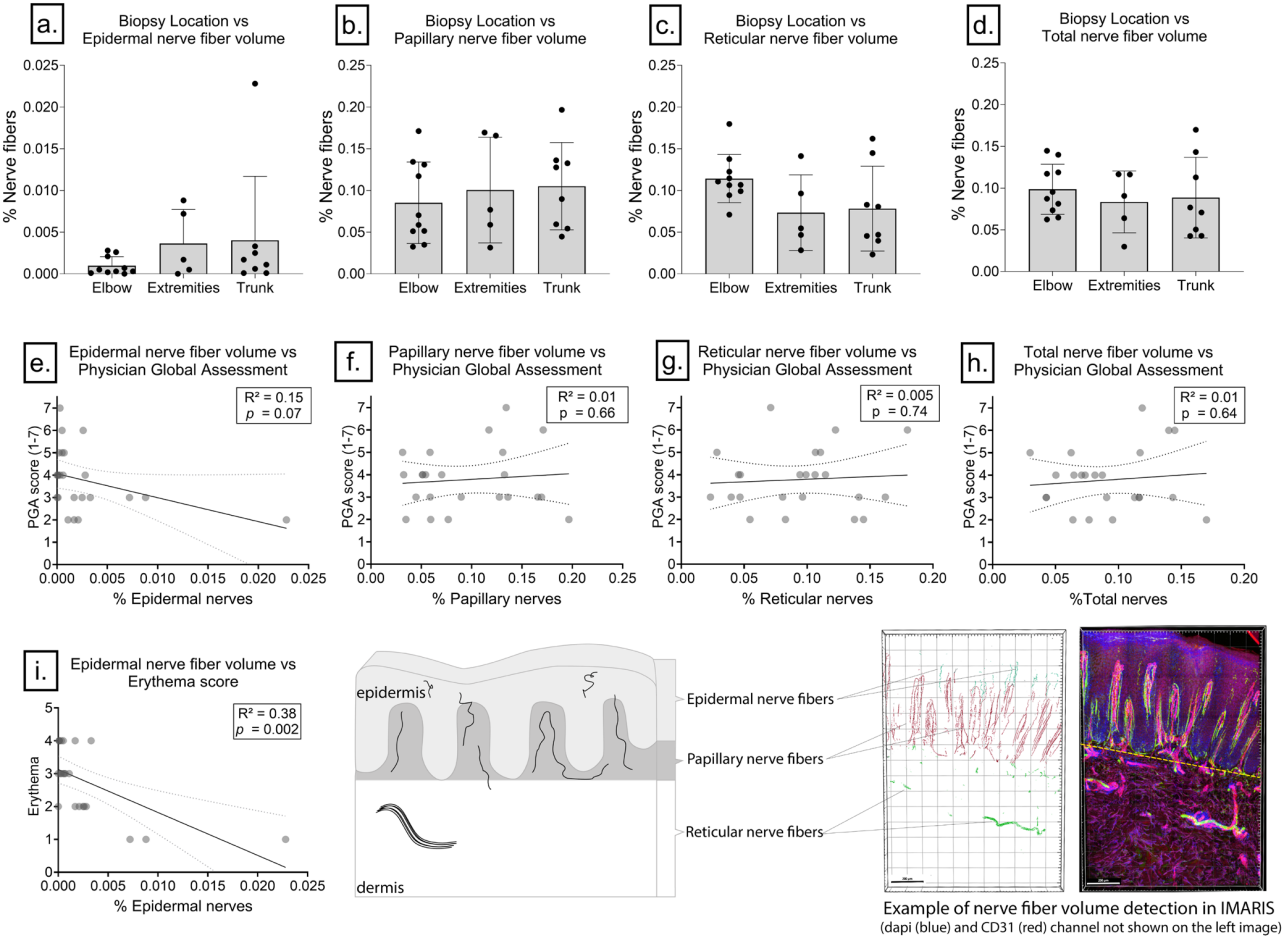
Nerve fibre volumes in different skin layers compared by biopsy location (elbow, extremities, trunk) and correlations with Physician Global Assessment (PGA). (a–d) Nerve fibre volume (%) in the epidermis (a), papillary dermis (b), reticular dermis (c) and total nerve fibre volume (d) are shown for different biopsy locations. No significant differences in nerve volume were observed between these locations. (e–h) Linear regression plots between nerve fibre volumes in the epidermis (e), papillary dermis (f), reticular dermis (g) and total nerve volume (h) with clinical severity as indicated by the Physician Global Assessment (PGA). Despite the trends, none of the correlations reached statistical significance (*p* > 0.05).

No significant differences in nerve fibre density were identified based on the biopsy location, which was divided into trunk, extremities and elbows (*p* > 0.05) (Figure 4a–d). Thus, the location of the biopsy does not explain the observed variation between patients. We also found that patients with family members who are also affected by psoriasis had a significantly thinner epidermis than patients without family members affected by psoriasis.

A correlation matrix was generated to explore potential correlations between nerve fibre density and (continuous) clinical parameters (Figure S2). Although clinical severity (PGA) appeared to exhibit a slight correlation with epidermal nerve fibre volume, no statistically significant Pearson's r or *R* ^2^ values were observed (*r* = −0.38, *R* ^2^ = 0.15, *p* = 0.071) (Figure 4e). The relationship between PGA and papillary nerves was even less strong (*r* = 0.1, *R* ^2^ = 0.01, *p* = 0.66), as was the relationship between PGA and reticular nerves (*r* = 0.07, *R* ^2^ = 0.005, *p* = 0.74), as well as the relationship between PGA and total nerve fibre volume (*r* = 0.1, *R* ^2^ = 0.01, *p* = 0.64) (Figure 4f,g). Thus, nerve fibre density has no relationship with the observed clinical severity of psoriatic plaques.

Significant correlations were observed between age and total nerve fibres (*r* = −0.46, *p* = 0.04), as well as between BMI and age (*r* = 0.68, *p* > 0.001). Although BMI did not correlate significantly with total nerve fibres (*r* = −0.35, *p* = 0.1), weight and height individually did correlate significantly with total nerve fibres. Significant correlations were also observed between epidermal nerve volume and erythema score (*r* = −0.62, *p* = 0.002). There was no significant correlation between itch, induration, smoking status and scaling with any of the nerve fibre volumes (*p* > 0.05).

## 4 Discussion

This study provides the most detailed 3D characterisation to date of nerve fibre distribution in psoriatic skin, highlighting the topographic organisation of cutaneous innervation and its spatial relationship to vascular structures and clinical parameters. By leveraging volumetric imaging of thick skin sections, we were able to delineate epidermal, papillary and reticular nerve networks with unprecedented resolution. Our results challenge simplistic notions of global nerve hyperplasia or hypoplasia in psoriasis and instead underscore the selective relevance of specific nerve subtypes, in particular epidermal fibres, in relation to local inflammation and clinical severity.

We found that epidermal nerve fibre volume, although representing the smallest fraction of total nerve density, exhibited the greatest inter‐patient variability and the strongest associations with clinical markers of inflammation. Specifically, epidermal innervation negatively correlated with erythema and epidermal thickness (both surrogate indicators of inflammatory activity) as well as with age. These correlations suggest that epidermal nerves may serve as structural correlates of neurogenic inflammation, potentially modulating vasodilation, mast cell degranulation and keratinocyte activation through neuropeptides such as CGRP, SP and VIP [4, 10, 11].

Importantly, we observed no significant associations between total or dermal nerve density and key clinical metrics such as Physician Global Assessment (PGA), itch or plaque morphology (erythema, induration, scaling). This finding implies that the presence of nerves per se is not sufficient to explain symptom severity and instead suggests that functional aspects, such as neuropeptide release frequency or receptor activation, are more likely to drive symptom expression. This is consistent with prior studies reporting both increased [13, 14] and decreased [15, 16] nerve densities in psoriasis, underscoring the importance of standardised 3D methods and careful anatomical stratification.

Our 3D reconstructions revealed that most epidermal nerve fibres did not arise directly from the reticular dermis, as commonly depicted, but instead branched off from perivascular networks in the papillary dermis. This architecture supports the hypothesis that neurovascular cross‐talk, rather than epidermal isolation, underpins key aspects of psoriatic inflammation. Although papillary nerves were abundant and intricately associated with capillaries, their volume did not correlate with vascular density, nor did we find meaningful associations between vascular volume and nerve density in any dermal compartment. Thus, anatomical proximity between nerves and vessels does not imply quantitative coupling, but may still reflect coordinated functional interactions mediated by neuropeptides [12, 19, 20].

Interestingly, nerve fibres were frequently observed near perivascular immune infiltrates. While not formally quantified, this co‐localisation suggests potential neuro‐immune interactions, particularly involving dendritic cells and macrophages capable of responding to nerve‐derived mediators. This aligns with mechanistic models proposing that neuropeptides enhance leukocyte recruitment, endothelial activation and IL‐23/Th17 signalling, which are considered central pathways in psoriasis pathogenesis [21, 22, 23, 24]. These interactions may form a positive feedback loop, wherein inflammation induces nerve growth via NGF, further amplifying neurogenic signalling [5, 25].

Contrary to some prior reports, we did not observe a correlation between itch and nerve fibre density. This discrepancy may stem from methodological differences in itch assessment or lesion chronicity, as earlier studies have shown that the relationship between innervation and itch is highly dependent on plaque age and neuropeptide expression patterns [26, 27, 28]. Notably, our dataset did not include information on lesion duration or uninvolved skin, which limits our ability to fully interpret changes over time or relative to baseline.

The high inter‐patient variability in epidermal nerve density, explaining over 75% of total variation, further supports the need for individualised assessments in future studies. Our findings suggest that static measures of nerve density may be insufficient as global biomarkers, but could prove valuable in treatment monitoring, especially for neuromodulatory interventions such as botulinum toxin or pulsed dye laser therapy. Moreover, the established 3D baseline anatomy offers a critical resource for biophysical modelling of light–tissue interactions, as demonstrated in our recent work on pulsed dye laser simulation [18].

Finally, this study highlights that the contribution of nerves to psoriasis is likely more functional than structural. Future research should integrate neuropeptide staining (e.g., CGRP, SP, VIP), nerve‐receptor staining, or functional nerve assessments to distinguish between active and quiescent neural components in psoriatic lesions. Combining 3D morphology with molecular and physiological data will be essential for identifying robust, mechanistically informed biomarkers of psoriasis pathogenesis.

## 5 Conclusion

In psoriatic skin, epidermal nerve fibres were significantly inversely associated with erythema but not with PGA‐assessed disease severity; total nerve volume was likewise unrelated to PGA. This points toward a compartment‐specific and functionally nuanced role of nerves in psoriasis. Our 3D dataset provides a foundational reference for future mechanistic and therapeutic studies targeting neuro‐immune interactions in psoriatic disease.

## Author Contributions

M.D. performed the experiments, analysed the data and wrote the manuscript. N.B., M.C.G.A., E.N.T.P.B. and T.G.L. supervised the process of the study. M.N. provided blinded PGA scores and expert opinions on the interpretation of the clinical data.

## Ethics Statement

This study was approved by the Medical Ethical Human Subjects Committee MEC‐U (Nieuwegein, The Netherlands) under registration number NL75576.100.20.

## Conflicts of Interest

The authors declare no conflicts of interest.

## Supporting information


**Data S1:** Supporting Information

## Data Availability

The data that support the findings of this study are available from the corresponding author upon reasonable request.
