# Supplementary material for: Nerve Fibres in Psoriatic Skin and Their Relation to Vasculature and Clinical Parameters
Source: Exp Dermatol. 2025 Sep 24;34(9):e70166. doi: 10.1111/exd.70166 (PMC12460965; doi:10.1111/exd.70166)
Supplement: Supplementary file 1 — Data S1: Supporting Information [file EXD-34-e70166-s001.docx]

**SUPPLEMENTS**


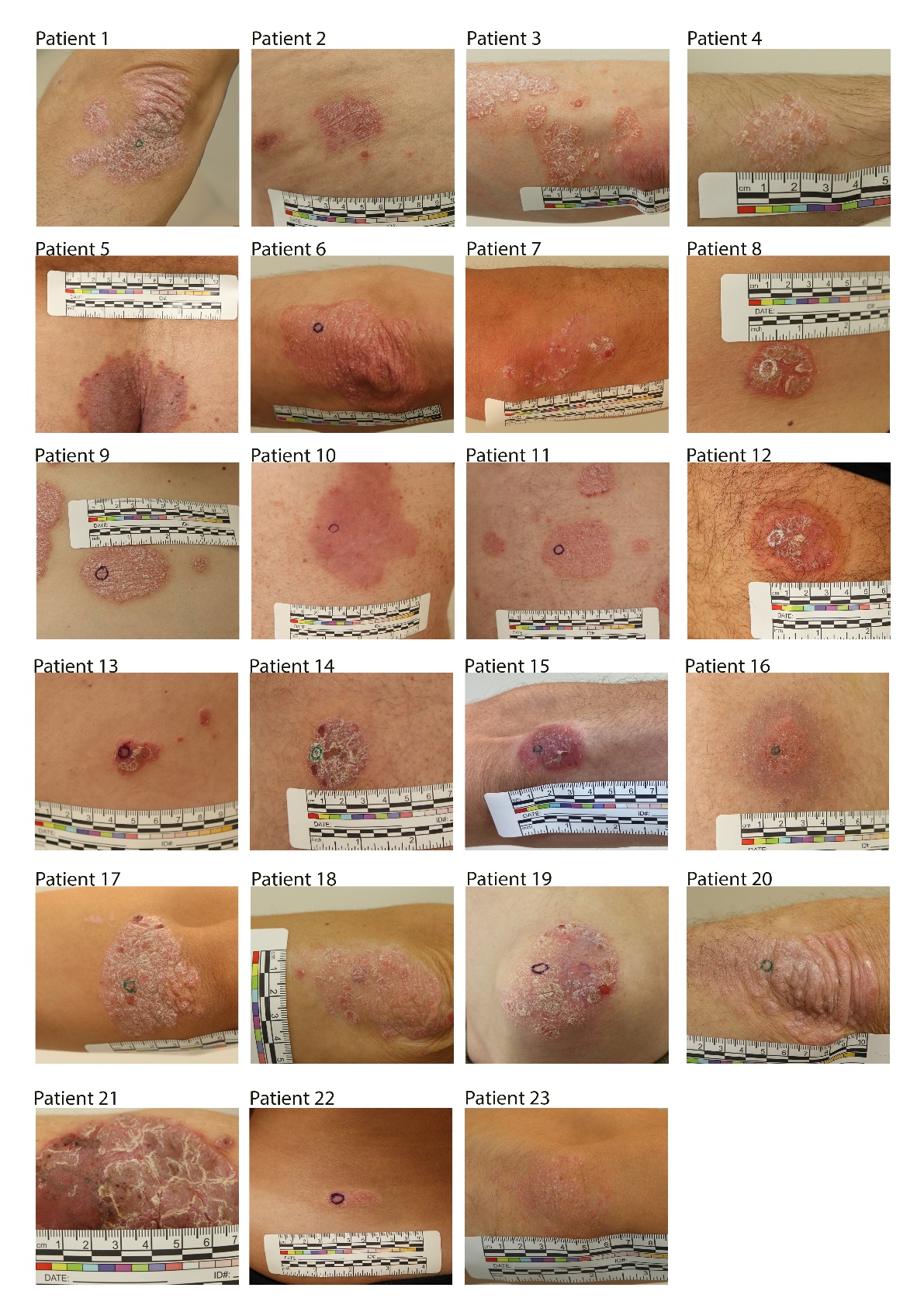


**Supplemental Figure 1** images of psoriatic lesions from 23 patients. Each image shows the severity and extent of erythema, scaling, and plaque thickness across different patients. Standardized measurement scales are placed next to each lesion for reference. Variations in plaque morphology are evident, highlighting the heterogeneity in the clinical presentation of psoriasis across individuals


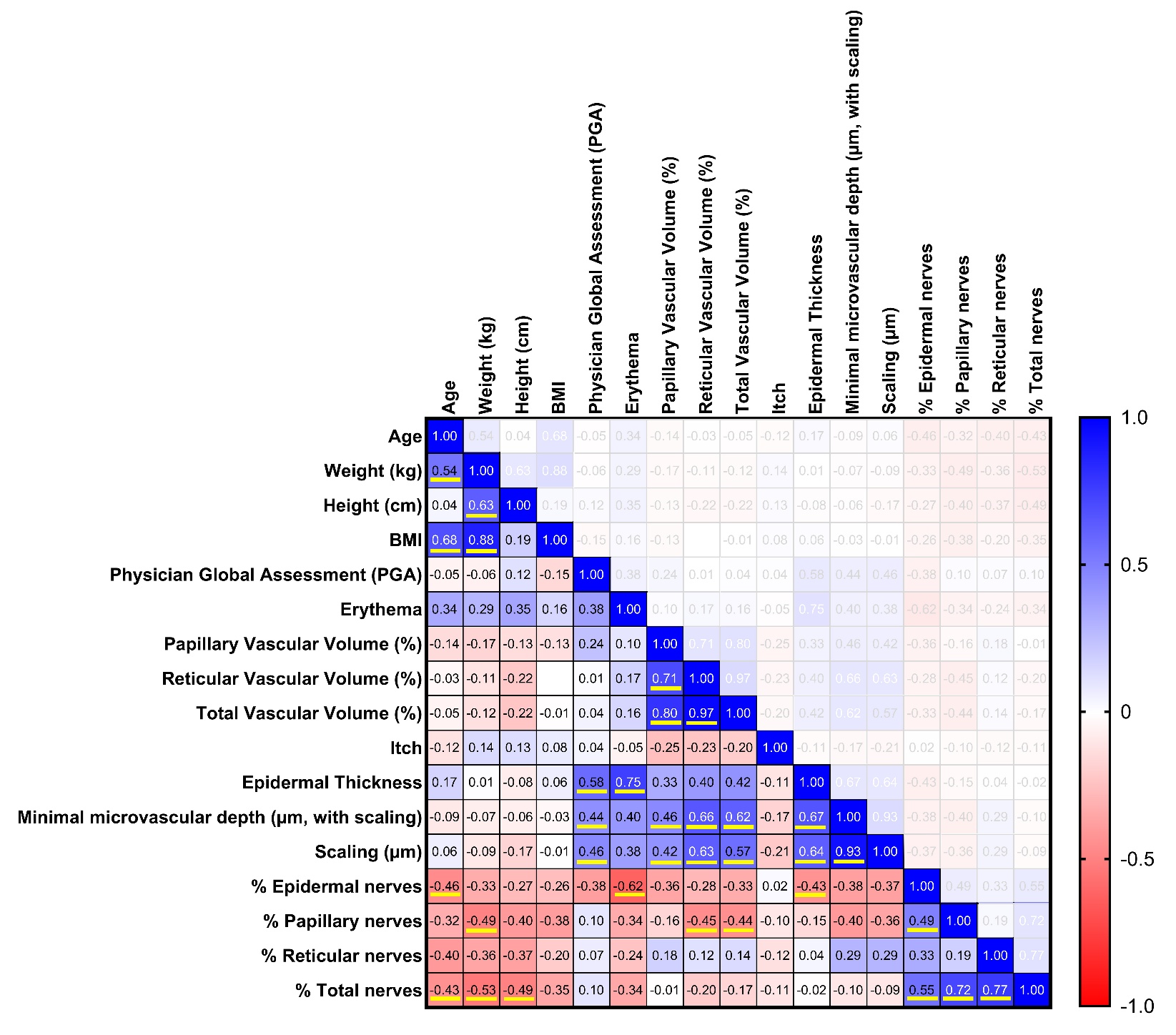


**Supplemental Figure 2** Correlation matrix depicting relationships between clinical and histological parameters in psoriasis. The heatmap illustrates the strength of correlations between various parameters, including age, weight, height, BMI, Physician Global Assessment (PGA), erythema, vascular volume, nerve fiber densities, epidermal thickness, and scaling. Blue shades represent positive correlations, while red shades indicate negative correlations. Stronger correlations are indicated by darker shades. Values shown are pearson r values. Significant correlations ( P<0.05) are highlighted with yellow underscore.

**Supplemental Table 1** Contribution of variation between nerve fiber volume for patients and sections

| Nerve fiber type | Variability explained by Patient (%) | F | *p* | Variability explained by Section nr (%) | F | *p* | Coefficient of variation |
| --- | --- | --- | --- | --- | --- | --- | --- |
| Epidermal nerve | 76.87 | 6.9 | <0.0001 | 0.83 | 0.82 | 0.45 | 190.4% |
| Papillary nerve | 80.11 | 8.24 | <0.0001 | 0.45 | 0.51 | 0.61 | 54.07% |
| Reticular nerve | 50.83 | 2.16 | 0.015 | 2.07 | 0.97 | 0.39 | 47.07% |
| Total nerve | 60.23 | 3.08 | 0.0007 | 0.62 | 0.35 | 0.71 | 40.80% |


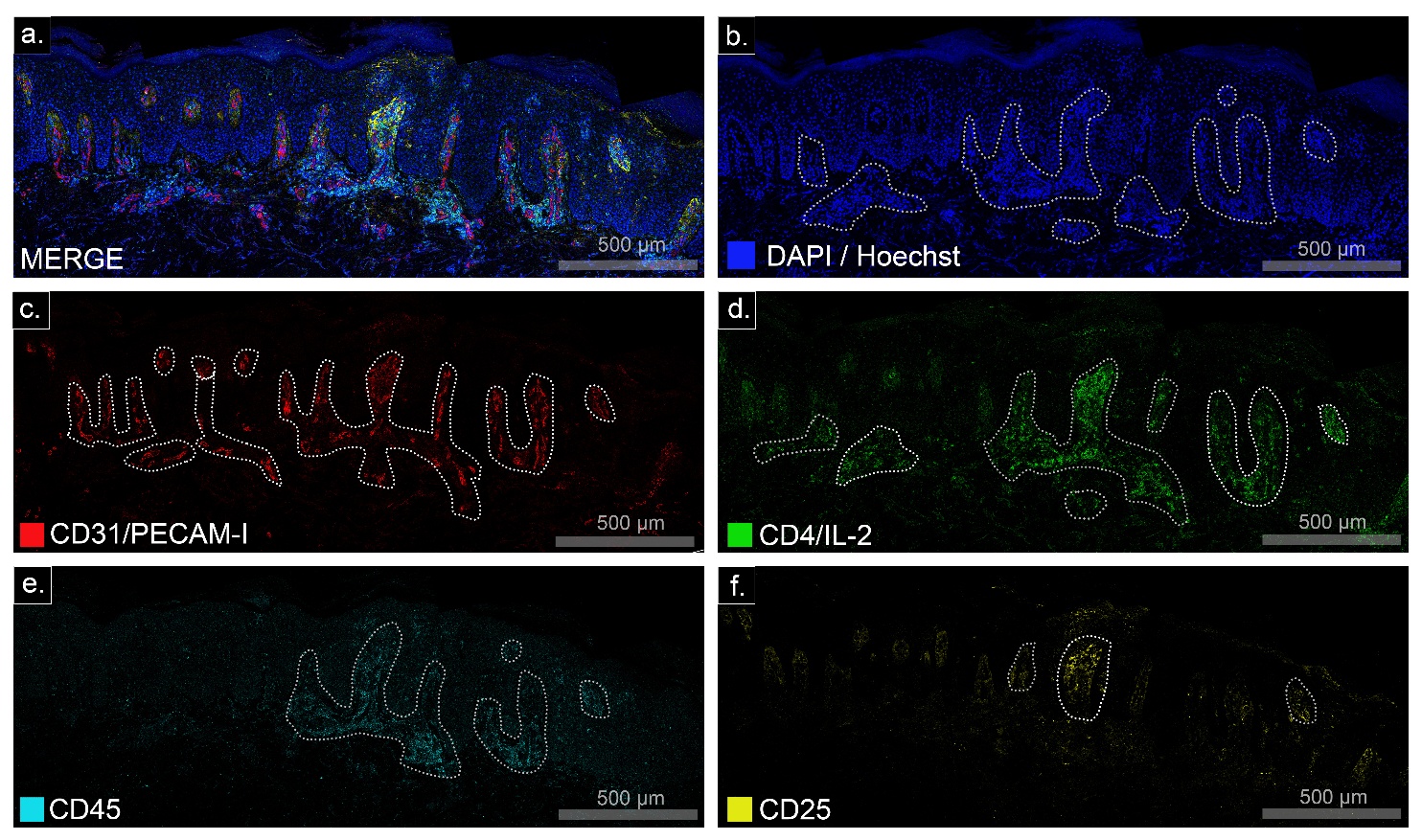


**Supplemental Figure 3** Immunofluorescent staining of psoriatic skin highlighting various immune markers.(a) MERGE: Composite image showing the overlay of all stained markers in the psoriatic lesion. (b) DAPI/Hoechst: Blue staining indicates cell nuclei. (c) CD31/PECAM-1: Red staining highlights endothelial cells associated with blood vessels. (d) CD4/IL-2: Green staining denotes CD4+ T cells suggesting immune cell activity. (e) CD45: Cyan staining represents leukocyte common antigen, marking immune cells. (f) CD25: Yellow staining shows the expression of the IL-2 receptor, associated with T cell activation. Dashed lines indicate regions of interest, including the dermal and epidermal layers. Scale bar: 500 µm.
